# Biosynthesis of ZnO, Bi_2_O_3_ and ZnO−Bi_2_O_3_ bimetallic nanoparticles and their cytotoxic and antibacterial effects

**DOI:** 10.1002/open.202300176

**Published:** 2024-01-17

**Authors:** Mina Sarani, Majid Darroudi, Mahin Naderifar, Majid Reza Akbarizadeh, Marcos A. L. Nobre, Benjamin Kruppke, Hossein Ali Khonakdar, Mehdi Erfani Jazi

**Affiliations:** ^1^ Department of Polymer Processing Iran Polymer and Petrochemical Institute Tehran Iran; ^2^ Department of Medical Biotechnology and Nanotechnology Faculty of Medicine Mashhad University of Medical Sciences Mashhad Iran; ^3^ Department of Basic Medical Sciences Neyshabur University of Medical Sciences Neyshabur Iran; ^4^ Faculty of Nursing and Midwifery Zabol University of Medical Sciences Zabol Iran; ^5^ Department of pediatric Amir Al Momenin Hospital Zabol University of Medical Sciences Zabol Iran; ^6^ São Paulo State University (Unesp) School of Technology and Sciences Presidente Prudente SP-19060-900 Brazil; ^7^ Max Bergmann Center of Biomaterials Institute of Materials Science Technische Universität Dresden 01069 Dresden Germany; ^8^ Department of Chemistry and Center for Photochemical Sciences Bowling Green State University Bowling Green OH-43403 USA

**Keywords:** ZnO−Bi_2_O_3_ NP, MCF-7, *Biebersteinia Multifida*, *S. epidermidis*, MTT

## Abstract

This work introduces an easy method for producing Bi_2_O_3_, ZnO, ZnO‐Bi_2_O_3_ nanoparticles (NPs) by *Biebersteinia Multifida* extract. Our products have been characterized through the outcomes which recorded with using powder X‐ray diffractometry (PXRD), Raman, energy dispersive X‐ray (EDX), field emission‐scanning electron microscopy (FE‐SEM), and Fourier‐transform infrared (FT‐IR) techniques. The finding of SEM presented porous structure and spherical morphology for Bi_2_O_3_ and ZnO NPs, respectively. While FE‐SEM image of bimetallic nanoparticles showed both porous and spherical morphologies for them; so that spherical particles of ZnO have sat on the porous structure of Bi_2_O_3_ NPs. According to the PXRD results, the crystallite sizes of Bi_2_O_3_, ZnO and ZnO−Bi_2_O_3_ NPs have been obtained 57.69, 21.93, and 43.42 nm, respectively. Antibacterial performance of NPs has been studied on *Staphylococcus epidermidis and Pseudomonas aeruginosa* bacteria, to distinguish the minimum microbial inhibitory concentration (MIC). Antimicrobial outcomes have showed a better effect for ZnO‐Bi_2_O_3_ NPs. Besides, wondering about the cytotoxic action against cancer cell lines, the MTT results have verified the intense cytotoxic function versus breast cancer cells (MCF‐7). According to these observations, obtained products can prosper medical and biological applications.

## Graphical abstract

In this work, attempted to arrange synthesized Bi_2_O_3_, ZnO, ZnO−Bi_2_O_3_ nanoparticles by *Biebersteinia Multifida* extract and then their cytotoxicity and anti‐bacterial investigate. This is a report on the synthesis and anticancer performance of synthesized samples on breast cancer cells (MCF‐7), as well as their anti‐bacterial activity against *Staphylococcus epidermidis* and *Pseudomonas aeruginosa* bacteria.

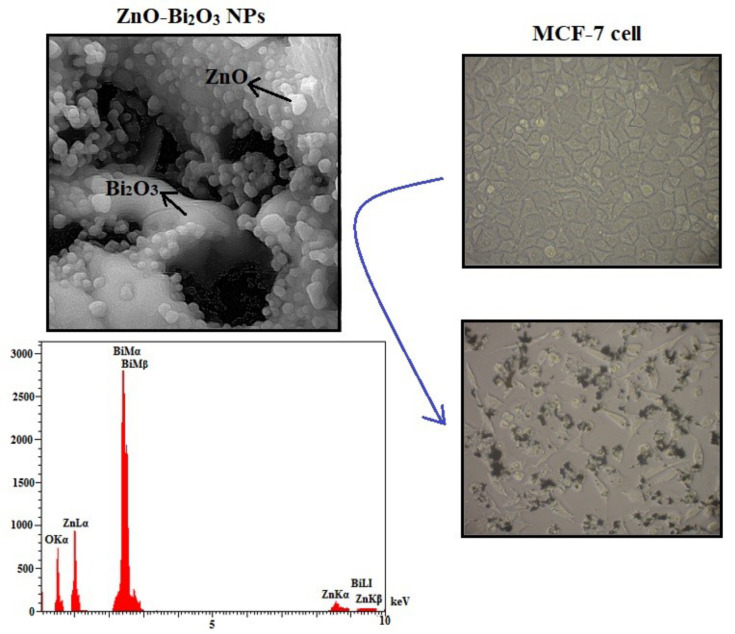

## 1 Introduction

Breast cancer can interfere with the cell division process of breast cells. These cells show an abnormal multiplication rate. There are varieties of breast cancer depending on type of affected breast cells, and it may start in different parts of the breast.[^1^, ^2^] Although, various treatment options are available, but each method has its side effect and disadvantage. For instance, the use of tamoxifen in cases of breast cancer can cause endometrial tissue cancer.[^2^, ^3^, ^4^, ^5^]

While traditional methods of breast cancer treatment destroy cancer cells, but also run the risk of damaging healthy tissue.^[6]^ Currently, scientists are studying nanotechnology‐based treatments to overcome this limitation and increasing the chance of survival for several cancers.[^7^, ^8^, ^9^] Nanotechnology enhances chemotherapy and it reduces side effects by directing drugs to breast cancer cells. It can also make surgery more precise and rectify the effectiveness of radiation therapy and another current therapeutic option. The outcome is that the risk to the patient is reduced and the chance of survival is increased.^[9]^ Researchers are using the newly discovered nanoparticles to develop new treatments which including new properties for medical science.[^10^, ^11^, ^12^, ^13^, ^14^, ^15^, ^16^, ^17^] Nanoparticles promote co‐administration, therapy and multimodal delivery (combination of therapy and diagnosis). The energy absorption and reflection properties of nanoparticles also enable them to improve the application of slow laser burns and hyperthermia, which can damage diseased tissues.[^18^, ^19^, ^20^, ^21^]

Nanomaterials have spatial feature because of their small size and high surface‐to‐volume ratio. Metal oxide nanoparticles are the focus of researchers because of their unrivaled physicochemical properties.[^22^, ^23^, ^24^, ^25^, ^26^, ^27^] Meanwhile, bismuth oxide is a good candidate of metal oxides with various applications. The special properties of these nanoparticles can expand their use in biological and medical fields.^[28]^ For example, the use of bismuth oxide nanoparticles in cancer radiotherapy and imaging has improved the level of medical science and the quality of treatment.^[29]^ Bismuth oxide has different polymorphs depending on the temperature conditions. Bismuth nanostructures can be prepared by different methods such as solution combustion, thermal solvent, water heating, microwave, and sol‐gel, pyrolysis, and thermal decomposition.[^30^, ^31^] Bismuth‐based compounds are widely utilized as pharmaceuticals to treat gastrointestinal disorders such as dyspepsia, stomach ulcers, and helicobacter pylori infections. Recently, its medical applications have been expanded to include potential therapeutics for viral infections, cancer, bio‐sensing as well as drug delivery.[^32^, ^33^, ^34^]

Zinc oxide nanoparticles have wide applications that can be used in various fields such as catalysts, biosensors, ceramics, photo detectors, plastics, cosmetics, antiviral agents in coatings, pigments, optical materials, cosmetics, photocatalytic, electronic optical systems, and wastewater treatment.[^35^, ^36^] Zinc oxide nanoparticles have a diverse group of growth morphologies such as rod, plate, belt, flower‐shaped nanostructures, etc., which each creates special properties in this material.^[36]^ There are diverse methods including physical, chemical and biological procedures for the product of this substance. In biological synthesis methods, preparing of metal oxide nanomaterials is done through plants or microbes, which are the final products that are biocompatible and can cause the stability of oxide nanoparticles by creating a coating. Although chemical synthesis is harmful to the environment, it has received more attention owing to better control over purity, stability, morphology, and particle size. Choosing an efficient synthesis process can play an essential role in efficiency of synthesized nanocrystal material by affecting the morphology of the material.[^37^, ^38^, ^39^]

Chemical reducing agents are cytotoxic agents. On the other hand, the synthesis of nanoparticles by chemical and physical methods is expensive. Therefore, the biological product of nanoparticles has been developed by living microorganisms or obtained cell extracts from them as an appropriate alternative to the method chemical and physical manners.^[40]^ Easy availability, nontoxic nature, various options available, environmentally friendly protocols, and a quick synthesis procedure have been made the green technique the best choice for nanoparticles synthesis.^[38]^

Synthesis of metal oxide nanoparticles *via* green agents has attracted lots of consideration owing to diverse optical, chemical, photo‐electrochemical and electronic attributes. The characteristics of produced nanoparticles by plant extracts depend on factors such as the nature of plant extracts, extract concentration, reaction temperature, reaction time, pH, metal salt concentration. In this method, plants containing antioxidants are used, which play an important role in absorbing free radicals.[^41^, ^42^] Many studies have represented that plant metabolites, carbohydrates and proteins are covering the surface layer of the produced NPs in this way. Besides stability, these elements lead to more biocompatible particles and their use in the biological area will be minor demanding.^[43]^

Although, toxicity of Bi‐based and bimetallic compounds has been studied for years, information on toxicity effects on cancer human cells is very little and there is serious concern about the side effects of these nanoparticles. The researcher of our team has investigated the influence of their synthesized nanoparticles on several cancer cell lines such as MCF‐7,^[44]^ U87,^[45]^ and MDA‐MB‐231.^[46]^ These results have been presented that our synthesized nanoparticles have a proper and acceptable cytotoxic impact on the studied cancer cell lines. Therefore, the target of this research is the green preparation and characterization of Bi_2_O_3_, ZnO, and ZnO−Bi_2_O_3_ nanoparticles using *Biebersteinia Multifida* and investigation of anti‐bacterial impact against *Staphylococcus epidermidis* and *Pseudomonas aeruginosa* bacteria, and following *in vitro* toxic effects of these nanoparticles on human breast cancer (MCF‐7) cells.

## Experimental

### Synthesis of Bi_2_O_3_ nanoparticles

The addition of distilled water and placing *B. Multifida* extract in a water bath at 80 °C to synthesize Bi_2_O_3_ changed the volume to 50 mL. Bi(NO_3_)_3_.5H_2_O (0.02 M, Merck) was added to the solution and mixed for 3 hours at 80 °C. The final solution was dried for 15 hours in an oven at 90 °C. Then it was calcinated in a digital furnace for 2 hours at 500 °C. It was demonstrated that the obtained orange precipitate is bismuth oxide nanoparticles (Bi_2_O_3_ NPs).

### Synthesis of ZnO nanoparticles

In order to provide the synthesis of zinc oxide nanoparticles, distilled water has been considered as solvent and the *B. Multifida* extract has been volumed using 50 mL of distilled water. After weighting, the extract solution was mixed with Zn(NO_3_)_3_.6H_2_O (0.01 M, Merck) at 80 °C for 3 hours. The final solution was dried in an oven for 15 hours at 90 °C. Then obtained product was calcinated in a digital furnace for 2 hours at 500 °C. It was verified that final product is zinc oxide nanoparticles (ZnO NPs).

### Synthesis of ZnO−Bi_2_O_3_ nanoparticles

Some distilled water was added to the *B. Multifida* extract and its volume reached 50 mL to synthesize NPs in two separate Erlenmeyer flasks. These two Erlenmeyer flasks were placed in a water bath at 80 °C. A solution was prepared with 1 : 2 ratio of Zn(NO_3_)_2_.6H_2_O (Merck) and Bi(NO_3_)_3_.5H_2_O (Merck). This solution was stirred for 2 hours at 80 °C and dried at 90 °C for 15 hours. At last, the dried sample was calcined in a digital furnace at 500 °C for 2 hours. It was proven that synthesized orange precipitate is bimetallic nanoparticles (ZnO−Bi_2_O_3_ NPs).

### Characterization

Different characterization procedures were applied to study the morphology, shape, and structure of synthesized NPs. Powder X‐ray diffractometry (PXRD, Netherlands, PANalyticalX'Pert PRO MPD system, Cu Kα) was applied to investigate the crystalline structure of synthesized NPs. The vibrational energy modes of a sample were analytically measured by Raman spectroscopy technique (Raman Takram P50C0R10 device, laser wavelength=532 nm), whilst distribution of particle size and surface structure and morphology were determined by FE‐SEM (MIRA3 TESCAN, Czech) analyses.

### Cytotoxic activity

The breast cancer (MCF‐7) cells were applied to measure the toxicity of synthesized nanoparticles. Cell lines were purchased from Pasteur Institute (Tehran, Iran) and they were cultured in high glucose DMEM (4.5 g/L) with 10 % (v/v) FBS, 100 units/mL penicillin, and 100 mg/mL streptomycin in a CO_2_ incubator (5 % CO_2_, 37 °C). Cells were seeded in tissue culture plates after reaching 80 % confluence. The next day, different concentrations of synthesized nanoparticles (20–5120 μg/mL) were added to the cells for 24 h at 37 °C, separately. After incubation, the MTT solution in PBS (5 mg/mL) was added into each well at 37 °C to incubate for three hours. The absorbance (Abs) of dissolved formazan precipitate was measured using a Stat FAX303 plate reader. The experiments were all repeated three times and the results were reported as mean±SD. The livability of treated cells was measured using the equation of Viability (%)=(Abs of treated cells/Abs of control)×100.

### Anti‐bacterial impact

In this study, the standard strains of *Staphylococcus epidermidis* (ATCC6538P) and *Pseudomonas aeruginosa* (ATCC15442) were used and accumulated from the microbial collection of the Scientific and Industrial Research Organization of Iran. Mueller Hinton Broth medium was used to survey the minimum inhibitory concentration (MIC) of samples. The culture medium was heated by a magnet on a heater stirrer, while stirring. After becoming clear, it was placed in an autoclave at 121 °C and a pressure of 15 Pascal for 15 minutes in order to be sterilized. First, 8 mg of each nanoparticle was weighed and dissolved in 2 mL of Mueller Hinton Broth medium. Then, serial dilutions (1–5120 mg/mL) were prepared. In following, 10 μL of the desired microbial suspension (equivalent to 0.5 McFarland×10^8^ CFU/mL) was added to each of the wells. The plates were incubated for 24 hours at 37 °C. After that, the growth of bacteria in the wells was determined to check the MIC of nanoparticles.

## 2 Results and Discussion

### 2.1 XRD

**Figure 1:**
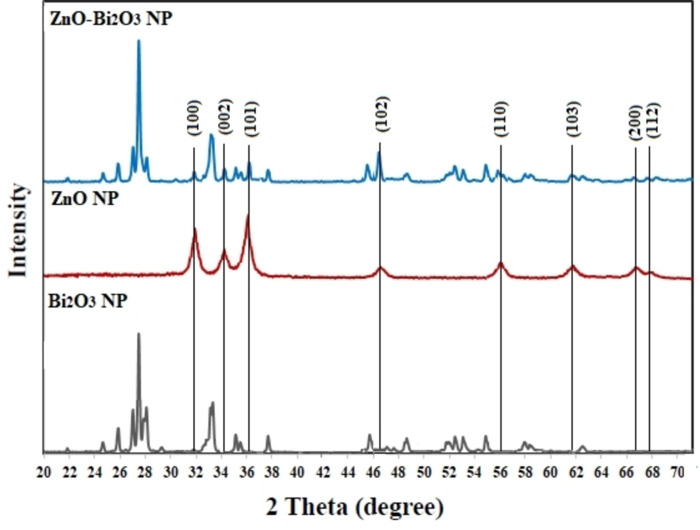
PXRD spectra of Bi_2_O_3_, ZnO and ZnO−Bi_2_O_3_ NPs.

The XRD spectra of Bi_2_O_3_, ZnO, and ZnO−Bi_2_O_3_ NPs are displayed in Figure 1. The main peaks of Bi_2_O_3_ NPs observed at 2θ values of 21.44, 24.62, 25.74, 27.21, 27.37, 28.16, 33.25, 35.40, 37.79, 40.27, 45.94, 46.51, 48.74, 52.57, 54.86, 57.85, and 63.43 which were in correspondence with standard code of JCPDS no. 41‐1449^[28]^ and be related to the monoclinic structure of Bi_2_O_3_. As seen in Figure 1, ZnO NPs displayed sharp diffraction peaks at 2θ=32.16, 33.12, 35.94, 48.08, 56.73, 63.58, 67.10, 68.15, 70.14, 73.10, and 77.12°, which were assigned to standard code of JCPDS card no. 36‐1451,^[32]^ indicating that hexagonal wurtzite structure for ZnO. The XRD pattern of bimetallic nanoparticles of both Bi_2_O_3_ and ZnO nanoparticles appeared, indicating the prosperous preparation of ZnO−Bi_2_O_3_ NPs (Figure 1). The crystallite size of nanoparticles was evaluated *via* Scherrer equation.^[39]^ The crystallite size of Bi_2_O_3_, ZnO and ZnO−Bi_2_O_3_ NPs was measured to be 57.69, 21.93, and 43.42 nm, respectively. The percentage crystallinity of synthesized nanoparticles was obtained using equation of % Crystallinity=(total area of crystalline peaks)/(total area of all peaks), and it was achieved 74.58, 86.22, and 69.76 % for Bi_2_O_3_, ZnO and ZnO−Bi_2_O_3_ NPs, respectively. In the case of the Specific surface area (SSA) can be calculated by Sauter formula (SSA=6000/p×D; where p is the density of synthesized nanoparticles and D is the size of particles), which was measured 49, 12, and 36 m^2^/g for Bi_2_O_3_, ZnO and ZnO−Bi_2_O_3_ NPs, respectively. As can show, SSA of Bi_2_O_3_ nanoparticles is higher comparted to ZnO and ZnO−Bi_2_O_3_ NPs.

### 2.2 SEM and EDX

**Figure 2:**
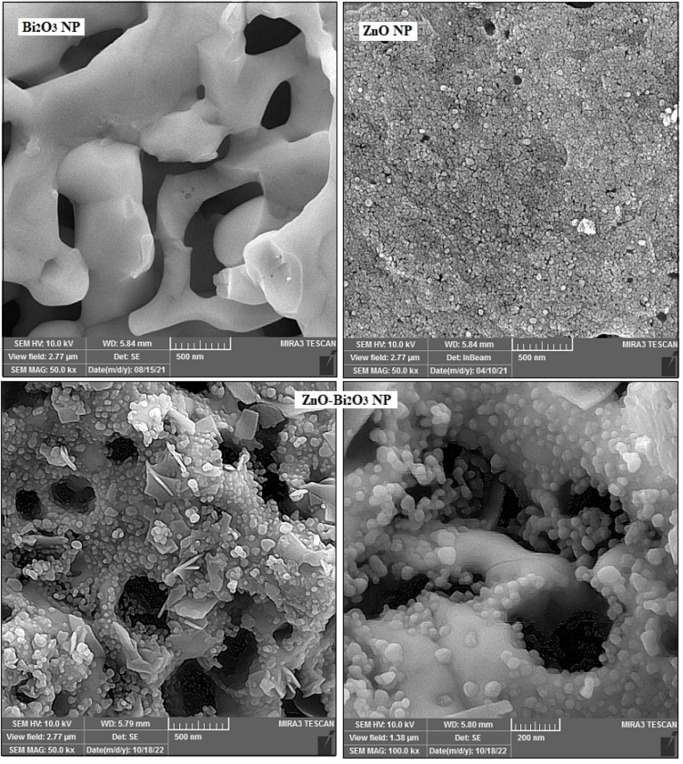
FESEM pictures of Bi_2_O_3_, ZnO and ZnO−Bi_2_O_3_ NPs.

**Figure 3:**
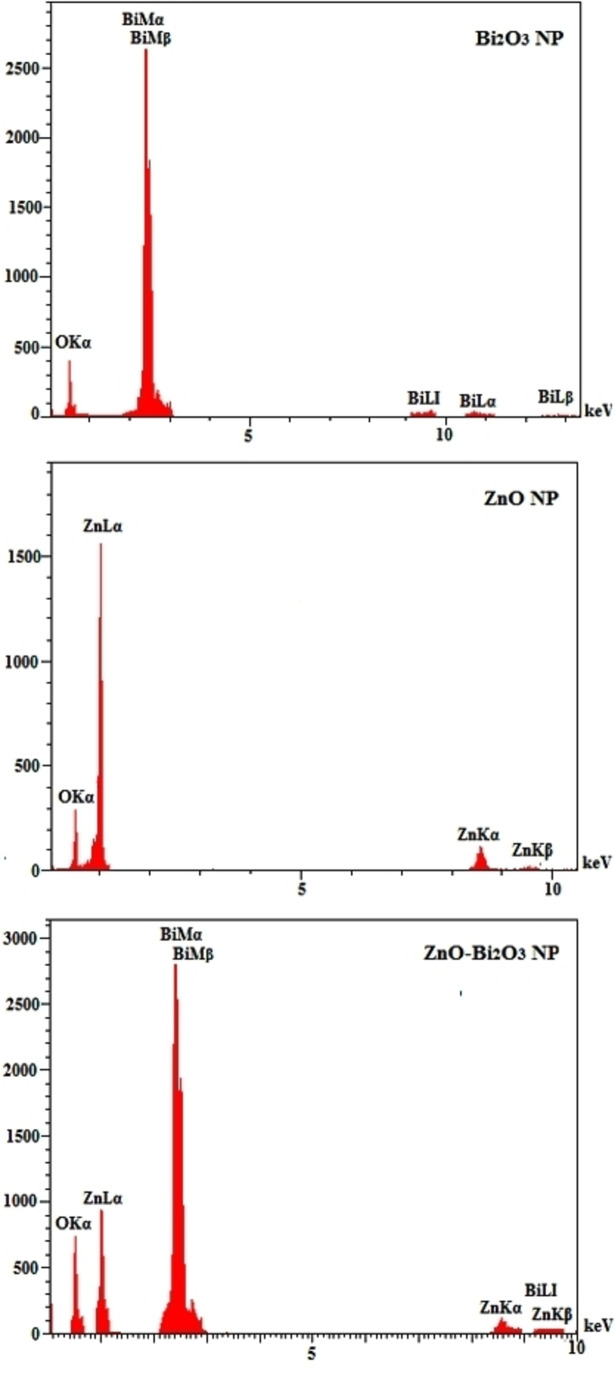
EDX patterns of Bi_2_O_3_, ZnO and ZnO−Bi_2_O_3_ NPs.

FE‐SEM, as a subcategory of the scanning electron microscope category, was applied to study the morphology and surface characteristics of various samples. Here, electron beams with particular wavelength and energy scavenge of sample surface. By using the data given from the detectors that collected the backscattered electrons from the sample surface, useful data was collected from sample surface. Figure 2 shows the FE‐SEM images of synthesized Bi_2_O_3_, ZnO, and ZnO−Bi_2_O_3_ NPs that obtained by using *B. Multifida*. As seen in Figure 2, Bi_2_O_3_ NPs have a porous structure and ZnO particles show a spherical morphology, while FE‐SEM image of bimetallic nanoparticles looks both porous and spherical in morphology. As can be observed in Figure 2, spherical particles of ZnO have sat on the porous structure of Bi_2_O_3_ NPs. One of the elemental analysis techniques is EDX microanalysis, which is based on generation of X‐rays in sample atoms through incident beam electrons. Figure 3 exhibits the EDX graphs of Bi_2_O_3_, ZnO, and ZnO−Bi_2_O_3_ NPs, which verified the attendance of elements in material structure. Therefore, the existence of bismuth and zinc elements all over nanoparticles is a confirmation of the successful synthesis of bimetallic NPs.

### 2.3 Raman

**Figure 4:**
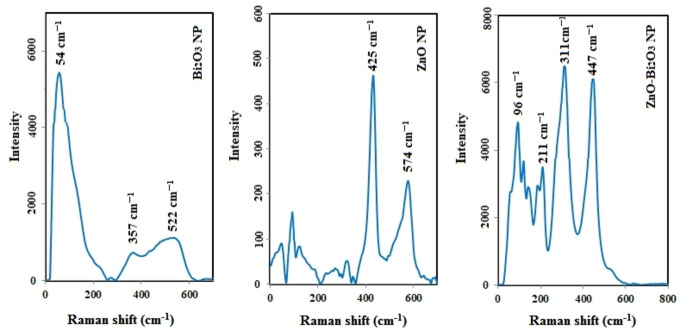
Raman graphs of Bi_2_O_3_, ZnO and ZnO−Bi_2_O_3_ NPs.

Raman is the survey of a type of interplay between light and matter which light undergoes inelastic scattering. In Raman spectroscopy, single wavelength photons (in the visible region) are focused on the sample. Photons interact with molecules, and then they are reflected, absorbed or scattered.^[47]^ Analyses are predicted 30 active Raman modes (15 Ag+15 Bg) for Bi_2_O_3_ monoclinic structure. The Raman spectra in Figure 4 demonstrate outstanding bands for sample, which matches with reported bands in the paper for Bi_2_O_3_ ^[28]^

The recorded band at 54 cm^−1^ is related to the bismuth vibration mode, whilst Bi−O scattering bands were recognized at 148 and 164 cm^−1^. Therefore, all of the other bands which are broader than 54, 148, and 164 cm^−1^ are attributed to oxygen vibration and represent a huge anharmonicity of oxygen vibration modes.

The spectrum has characteristic peaks around 379, 425, and 574 cm^−1^, which correspond to the ground phonon states of A_1_ (TO), E_2H_, A_1_ (LO), and E_2_ on hexagonal ZnO.^[40]^ The polar branches of A_1_ (TO) and A_1_ (LO) are appeared at 379 cm^−1^ and 574 cm^−1^. The A_1_ (LO) phonon state is related to the oxygen vacancies, and zinc interstitials or defect in ZnO structure. Becoming peaks around 75 cm^−1^ could be allocated to ground phonon states (E2L) of hexagonal ZnO (Figure 4). As seen in Figure 4, the intensity of peaks has increased in bimetallic graph, and the peaks relevant to ZnO and Bi_2_O_3_ can be shown in this graph as well. In Xin *et al*. research (2018), similar results have been presented on issues related to the bimetallic indium‐silver nanostructure.^[48]^

### 2.4 FT‐IR

**Figure 5:**
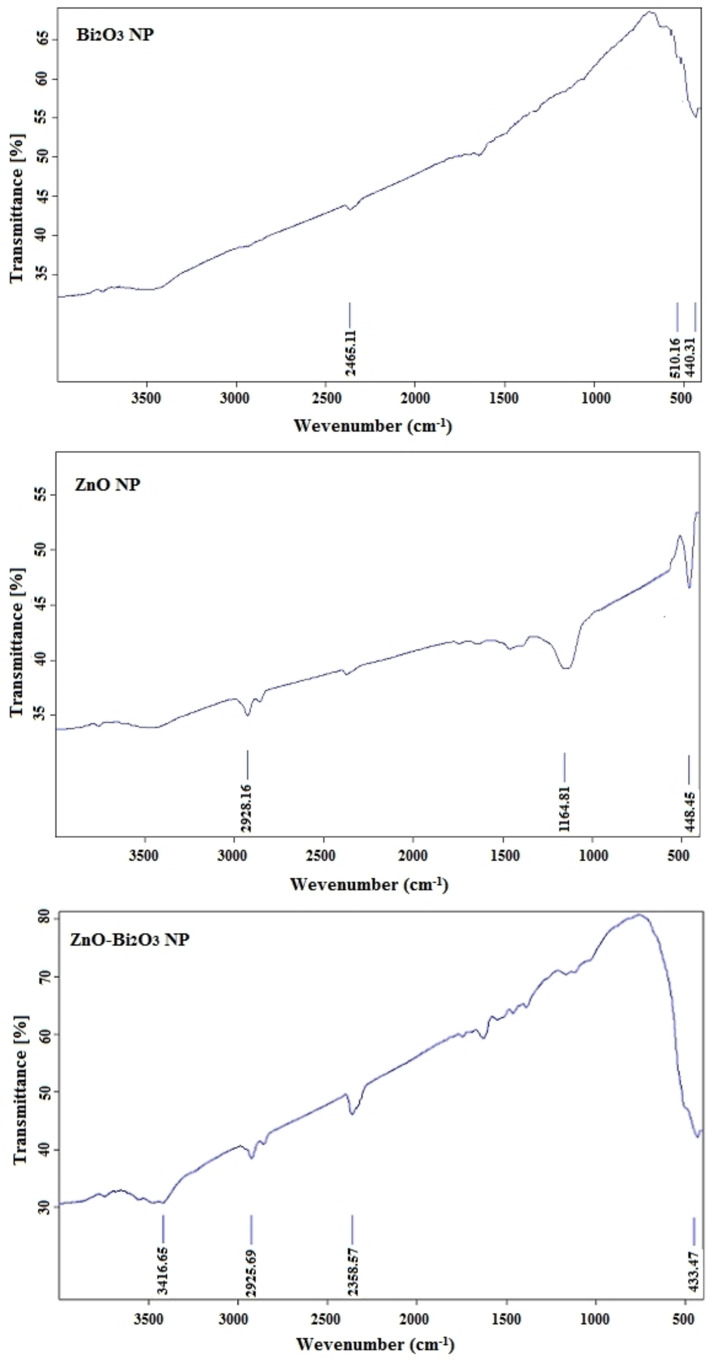
FT‐IR graphs of Bi_2_O_3_, ZnO and ZnO−Bi_2_O_3_ NPs.

Fourier Transform Infrared Spectroscopy (FTIR) is identified the Infrared absorption of chemical bonds in a molecule.^[40]^ This spectrum shows a distinct molecular fingerprint, which is very useful for characterizing covalent bond information and recognizing functional groups. Figure 5 displays the FT‐IR of Bi_2_O_3_, ZnO, and ZnO−Bi_2_O_3_ NPs. As seen in this Figure, the broad OH peak appeared in the ~3420 cm^−1^ region, which can be attributed to the OH stretching vibration. The peak at ~2460 cm^−1^ probably related to C−C and C−H stretching modes. In addition, appearance of two peaks of Bi_2_O_3_ at 440 cm^−1^ and 510 cm^−1^ is related to stretching vibration of Bi−O in Bi_2_O_3_; this peak was also mentioned in the research of Ai *et al*.^[49]^ Visible mode of the Bi−O−Bi stretching vibration at 618 cm^−1^ is in well consent with previous studies.^[50]^ According to Figure 5, the vibration mode of Zn−O at 448 cm^−1^ confirmed the synthesis of ZnO NPs.^[39]^ In the FT‐IR spectrum of bimetallic NPs, the bands of Bi_2_O_3_ and ZnO NP were observed in the 400–600 cm^−1^ region.

### 2.5 Anti‐bacterial effect

Nowadays, researchers have carried out a plenty of efforts to find new materials with significant antimicrobial properties which caused the progress of novel therapy options. Between these researches, nanomaterials appeared as considerable and new factors.^[51]^ So here, the anti‐bacterial of Bi_2_O_3_, ZnO and ZnO−Bi_2_O_3_ NP was studied through broth dilution method. Results of MIC are shown in the Table 1. According to the table, the highest inhibitory activity of nanoparticles was observed against *S. epidermidis* bacteria. Antimicrobial effects were obtained against *P. aeruginosa* at higher concentrations. As seen in Table 1, ZnO‐Bi_2_O_3_ NPs have shown a better antibacterial effect than Bi_2_O_3_ and ZnO nanoparticles.

**Table 1:** MIC of Bi_2_O_3_, ZnO and ZnO−Bi_2_O_3_ NPs to inhibit *S. epidermidis* and *P. aeruginosa*.

Nanoparticles	bacteria	
	*S. epidermidis*	*P. aeruginosa*
Bi_2_O_3_	320 μg/mL	5120 μg/mL
ZnO	160 μg/mL	1280 μg/mL
Bi_2_O_3_−ZnO	80 μg/mL	320 μg/mL

Several studies have presented that Bi_2_O_3_ NPs have good antibacterial impact on diverse pathogens *in vitro* and they have illustrated disparate anti‐bacterial mechanisms for Bi_2_O_3_ NPs, such as disturbing cell wall cytoplasmic membrane, and decomposing DNA.^[52]^ According to the conducted studies, regarding the possible antibacterial mechanism of zinc oxide nanoparticles, it can be stated that bacterial cells disturbed then tangency with zinc oxide NPs and their membranes are disrupted.^[53]^ It can be noticed that the membranes of bacterial cells are destroyed, and then tangency with NPs of zinc oxide. A study by Ramani *et al*. represented antibacterial properties of different structures of zinc oxide nanoparticles on four gram positive and four of gram‐negative bacterial; and their spherical shape ones were better antibacterial properties.^[54]^

### 2.6 Cytotoxic performance

**Figure 6:**
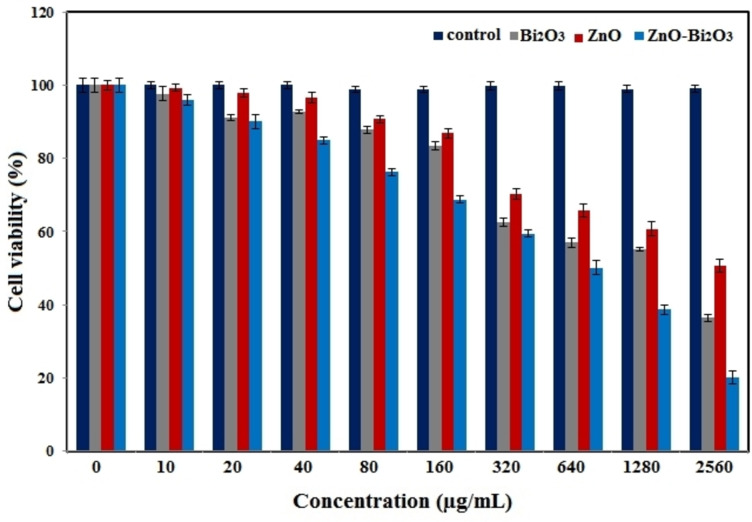
The cytotoxic activity of Bi_2_O_3_, ZnO and ZnO−Bi_2_O_3_ NPs against breast cancer cell (MCF‐7) line.

One of the topics of this research was to survey the cytotoxicity of Bi_2_O_3_, ZnO, and ZnO−Bi_2_O_3_ NPs in breast cancer (MCF‐7) cell line through MTT assay. Based on obtained outcomes, the bimetallic nanoparticles persuade a remarkable toxicity impact on MCF‐7 cell (Figure 6). It seems that increasing the concentration, the toxicity effect of nanoparticles increased. Since zinc oxide and bismuth oxide have different natures, the toxicity is different too. Therefore, it can be concluded that hybrid form of nanoparticles has extra or more properties than its metal oxide form. So here, ZnO−Bi_2_O_3_ nanoparticles, as hybrid form of ZnO and Bi_2_O_3_ nanoparticles, have showed more cytotoxic impact compared to ZnO nanoparticles and Bi_2_O_3_ nanoparticles form.

**Figure 7:**
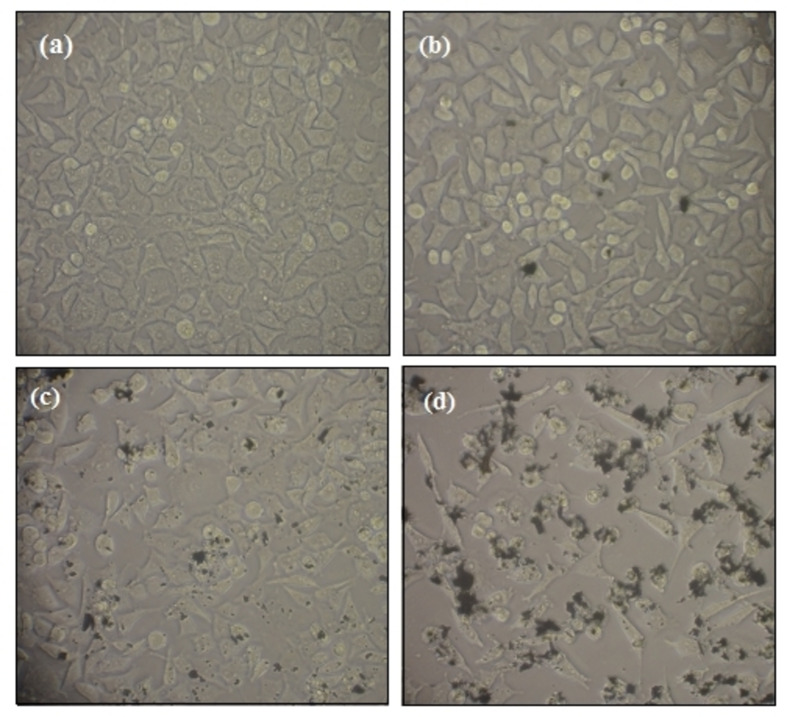
Illustration of breast cancer cell (MCF‐7) line treated with ZnO−Bi_2_O_3_ NPs at (a) control (b) 40 μg/mL (c) 320 μg/mL and (d) 2560 μg/mL concentration.

Figure 7 illustrates the cell before and after treatment with ZnO−Bi_2_O_3_ NPs. In addition, effect of NPs against MCF‐7 is perspicuously detectable. Considering the obtained data, increasing the concentration of NPs led to an increase in their lethal purpose.

Generally, a raise in NPs concentration accommodated more chance for their entrance into the cells in order to persuade their toxic impacts through making oxidative stress inward the cells, which causes the annihilation of cancer cells.^[55]^ N.O. Alafaleq *et al*. studied the anticancer activity of synthesized Cu−Mn bimetallic NPs on colon adenocarcinoma cancer (HT‐29) cell line. Their results presented that Cu−Mn bimetallic NPs treatment inhibited the destruction of cancer cells through DNA damage.^[56]^ In another study, Y. Cao *et al*. presented the anticancer property of green synthesized ZnO−CuO nanoparticles against lung cancer cell (A549) and human melanoma cancer cell (A375) lines. Based on the results, ZnO−CuO nanoparticles showed better cytotoxic impact on A375 compared to A549 cancer cells, which is due to the present of cupper metal in NP structures.^[57]^ Totally, the outcomes of this study confirm the remarkable lethality of synthesized NPs on MCF‐7 cells. In that matter, we proposed to importance of further survey on the biological attributes of these NPs for the suitability of the resultant as a hopeful anticancer compound for using in pharmaceutical centers.

## 3 Conclusions

The goal of this research was to present an easy and efficient synthetic way to produce Bi_2_O_3_, ZnO, and ZnO−Bi_2_O_3_ NPs through the usage of *B. Multifida* extract. The attributes of produced NPs were characterized by many techniques. The structure of NPs was supported by FE‐SEM images, which showed that Bi_2_O_3_ NPs have a porous structure and ZnO particles have a spherical morphology, while FE‐SEM images of bimetallic NPs appeared both porous and spherical morphology. According to the PXRD results, the crystallite sizes of Bi_2_O_3_, ZnO and ZnO−Bi_2_O_3_ NPs were 57.69, 21.93, and 43.42 nm, respectively. Antibacterial activity of synthesized NPs was surveyed on *Staphylococcus epidermidis* and *Pseudomonas aeruginosa* bacteria, and their antimicrobial results showed an acceptable effect for ZnO−Bi_2_O_3_ NPs. The cytotoxic potential of NPs on breast cancer cells was evaluated using MTT method. Based on the obtained findings, bimetallic NPs make a remarkable toxicity impact on MCF‐7 cell. Hence, the formulated NPs can be suggested a potential candidate for progressive medical goals.

## 4 Availability of data and materials

The present research outcomes have not been published before. Data and Materials are all in the main text, figures and tables.

## Conflict of interests

The authors declare that they have no competing interests.

5

## Data Availability

The data that support the findings of this study are available from the corresponding author upon reasonable request.
